# Phosphorus Fertilizers Enhance the Phytoextraction of Cadmium through *Solanum nigrum* L.

**DOI:** 10.3390/plants11030236

**Published:** 2022-01-18

**Authors:** Arosha Maqbool, Muhammad Rizwan, Tahira Yasmeen, Muhammad Saleem Arif, Afzal Hussain, Asim Mansha, Shafaqat Ali, Huda Alshaya, Mohammad K. Okla

**Affiliations:** 1 Department of Environmental Sciences and Engineering, Government College University, Faisalabad 38000, Pakistan; aroshamaqbool@gmail.com (A.M.); mrazi1532@yahoo.com (M.R.); rida_akash@hotmail.com (T.Y.); msarif@outlook.com (M.S.A.); afzaalh345@gmail.com (A.H.); 2 Department of Environmental Sciences, The University of Lahore, Lahore 54590, Pakistan; 3 Department of Chemistry, Government College University, Faisalabad 38000, Pakistan; 4 Department of Biological Sciences and Technology, China Medical University, Taichung 40402, Taiwan; 5 Cell and Molecular Biology Program, University of Arkansas, Fayetteville, NC 72701, USA; hmalshay@uark.edu; 6 Botany and Microbiology Department, College of Science, King Saud University, Riyadh 11451, Saudi Arabia; Malokla@ksu.edu.sa

**Keywords:** cadmium, phosphorus-fertilizer, oxidative stress, antioxidants, phytoextraction

## Abstract

Cadmium (Cd) toxicity strongly influences plants growth and seed germination in crop plants. This pot trial had aimed evaluate the benefits of two different kinds of phosphorus (P)-fertilizer in the phytoremediation of Cd by *Solanum nigrum* L. The current pot experiment was conducted to evaluate the role of P-fertilizers in phytoremediation of Cd by *Solanum nigrum* L. Single superphosphate (SSP) contain 7 to 9% P and Di-ammonium Phosphate (DAP) contain 46% P had been applied in single and combine form in soil with different ratios (0:0, 100:0, 0:100, 50:50%) accompanied by diverse Cd levels (0, 25, 50 mg kg^−1^). Three weeks seeding were transferred into pots, and plants had been harvested afterward seventy days of growth in the pots. Significantly inhibited plant growth was observed in shoots and roots of Cd contaminated plants. Cadmium stress had stimulated oxidative stress in subjected plants. However, supplementation of P-fertilizers in an optimum manner significantly increased plant biomass along with enhancing antioxidants enzymatic activities and inhibiting oxidative stress. Maximum plant-growth had been noted in SSP + DAP supplemented plants in contrast to single SSP, DAP supplemented plants. Higher Cd concentrations observed in SSP + DAP supplemented plants over single treatment. It has been concluded that combination of SSP + DAP might be a better option to improve growth as well as uptake capacity of *Solanum nigrum* L. under Cd stress. However, a field study is recommended for detailed future investigations.

## 1. Introduction

The world agriculture lands have to face unprecedented challenges since the industrialization, overpopulation as well as urban sprawl. The annual worldwide loss of fertility and productivity of land has increased many folds quickly [1]. Agriculture and industry have been considered the two key sources of soil depletion as well as deterioration. Agriculture and industry both are facing competition for the same natural resources which causes the manipulation of natural resources that leads environmental and soil contamination with heavy metals [2]. For croplands (Cd) is the most toxic and potent heavy metal due to its high solubility among others [3]. Unfortunately, Cd has a persistent and inorganic nature that does not undergo chemical or microbial degradation [4]. Soil is the starting point that is directly connected with the food chain. Food security and safety can be improved by food quantity and quality [2]. Croplands have significant Cd accumulation that has to have negative implications, potential health danger, as well as destructive consequences on the agro-ecosystem. For that reason, it is essential to investigate the Cd transport and buildup in the soil medium to plant system.

Cd in the plant system reacts as the strong inhibitor of photosynthetic activity [5]. Chlorophyll (Chl) loss and seizure of biosynthetic machinery subsequently disturbs enzyme activities, particularly Rubisco, which is most sensitive [6], inhibits Chl biosynthesis, disrupts chloroplast metabolism and hampers photochemical and carboxylation reactions of photosynthesis [7]. Typically, conventional physicochemical technology is most costly and disrupts the physical and chemical properties of the soil [8]. Phytoremediation is green approach to get rids of the pollutants and decontaminate the soil [9]. This technique has several advantages, mainly including autotrophic system with fast growing plants [10] that need a small amount of nutrients, are easy to manage, have aesthetic value, environmental sustainability, and are ecofriendly as well as cheap [11,12]. Phytoextraction may employ a hyper accumulator to extract heavy metal from polluted soil, which is observed as a possible remediation tools for Cd-contaminated soil because of its low cost and ecofriendly nature [13,14].

*Solanum nigrum* L. are a hyper accumulating plant species which have the ability to grow fast with high biomass even under stressful environmental conditions. This grows widely in South Asia and its juice is used for the cure of certain types of diseases [9]. It has excessive Cd Phytoextraction capability compared to other hyper accumulators such as *Sedum plumbizincicola* and *Noccaea caerulescens* [5]. However, higher Cd levels have negative effects on *S. nigrum* growth, they need a few adaptations, particularly at initial growing stages, to increase the phytoextraction and sustaining ability. Phosphorus (P) is one of the most indispensable macro elements after nitrogen (N). Adequate P fertilizer supplementations would be an effective approach to increased crop production and attain optimum yields [15,16]. P application have potential to change the bioavailability of heavy metals in soils [17]. Afzal et al. [18] described that P supplementation in paddy rice significantly decrease the Cd concentration while Gao et al. [19] reported that P fertilizer’s supplementations decrease the soil pH subsequently increases the accessibility of Cd. When P come in contact with Cd as a result Cd-P compounds, Cd_3_(PO_4_)_2_ is formed which might alleviate the toxicity of Cd [20], decline the accumulation of Cd in plants [21], and promote plant growth [18]. Therefore, we conceded this research to assess the interaction of P-Fertilizer and Cd contamination along with understand the mechanism of hyper accumulator plant *S. nigrum*.

It was hypothesized that P-fertilizers may enhance the phytoextraction of Cd by *Solanum nigrum* L. The current pot experiment was planned to evaluate the role of different kinds of (P)-fertilizers in phytoremediation of Cd by *Solanum nigrum* L. Overall the effects of different P-supplementation in the soil was observed on *S. nigrum* by measuring dry biomass of roots and shoots, chlorophyll contents, antioxidants enzymes and oxidative stress markers. This study was conducted to find a best concentration of P-fertilizers that would be effectively in phytoremediation of Cd polluted soil through *S. nigrum*.

## 2. Material and Methods

### 2.1. Experimental Design and Treatments

In this experiment soil had been collected from an agricultural field that was located in University of Agriculture Faisalabad (N 31°25′46.8048″, E 73°4′14.3112″). For soil collection, stainless steel blade had been used, then soil was mixed thoroughly and removed large debris and roots. After that, dried under shade, then 2 mm sieve had been used for sieving. Soil was sandy loam with silt, sand, and clay of 14, 68, and 17%, respectively. Soil had been categorized as having pH 7.32 (1:2.5 soil to water ratio); EC 1.83 dS m^−1^ and organic matter 0.64%. Total and available P concentrations were 2.20 mg kg^−1^ and Zn, Cu, Cd total and available concentrations were 0.81 mg kg^−1^; 0.34 mg kg^−1^ and 0.09 mg kg^−1^, respectively. Complete physicochemical characteristics of the soil used in this experiment are described before (Arosha et al., 2020) [22]. This experiment had been carried out in botanical garden of Government College University Faisalabad (Faisalabad, Pakistan; 31°25′0″ N 73°5′28″ E) during (April–June) in 2019. The seeds of *Solanum nigrum* L. had been acquired from the Ayub Agricultural Research Institute Faisalabad, Pakistan. The soil had been thoroughly mixed and spiked with discrete Cd (CdCl_2_ 2.5H_2_O) levels (0, 25, 50 mg kg^−1^). Two different kinds of phosphorus fertilizers Single superphosphate (SSP) and Di-ammonium Phosphate (DAP) had been added in the soil with varying ratios (0–0, 100–0, 0–100, 50–50 mg kg^−1^). P-fertilizers had been selected and obtained from the market. The concentrations of, Zn, Cd, and Cu had been fundamentally low and unnoticed in used fertilizers [23].

### 2.2. Soil Pot Experiment Design

In this work the *S. nigrum* seeds had been sterilized with 5% sodium hypochlorite (NaClO) for 10 min, wiped with faucet water four times, and afterward seeds had been soaked in deionized water for 6 h. The quartz sand plastic trays had been used for planting the seeds. Half strength Hoagland nutrient solution had been used for irrigation. Uniform seedlings had been transferred into pots (four seedlings for each pot) following 3 weeks of germination. All (every-one) pots had filled up with 5 kg soil. Each and every pot had been lay down in a nursery with completely randomized design with four replicates of every treatment while the tap water had been used to keep up 70% soil water-holding.

### 2.3. Harvesting Plant

After 70 days of sowing, plants had been harvested and carefully separated into shoots and roots. The root as well as shoot length, leaf width, number of leaves per pot, fresh weight had been measured. The measurements of root and shoot length had been measured with centimeter scale. The diluted acid of (HCl 1.0%) had been used to avoid the dirt evacuation on the surface after rinsed with deionized water three times. After this these all samples had been kept for drying up for 72 h at 70 °C. These dried plants had been sieved through 2 mm sieve mesh strainer for further analysis.

### 2.4. Assessment of Chlorophyll, Carotenoid Content and Photosynthetic Parameters

The sample of fresh leaf (0.5 g) had been dipped in acetone (85%, *v*/*v* Sigma) then supernatant had kept in dark place and centrifuged at (4000× *g* for 10 min, 4 °C) and readings had been had by spectrophotometer with wavelength (470, 647, and 664.5 nm). The evaluation of chlorophyll (Carotenoid and Chlorophyll a and b) contents had been carried out using equation [24]. Infra-Red Gas Analyzer (IRGA, LCA-4, Analytical Development Company, Hoddesdon, UK) was used for the assessment of stomatal conductance, transpiration rate, photosynthetic rate and water efficiency at sunny days (10:00 to 11:00 a.m.).

### 2.5. Assessment of Antioxidant Enzymes and Oxidative Stress Markers

After 70 days of sowing, measurement of both the antioxidants enzymes and oxidative stress markers had been conducted. Shoots sample were kept in glass tube vertically then heated at 32 °C for 2 h in known volume of distilled water for electrolyte leakage and this solution labelled as EC_1_ (electrical conductivity), after that same solution heated again for 20 min at 121 °C temperature EC_1_ was measured later cooling down at room temperature EC_2_. The EL (electrolyte leakage) was calculated by the help of this equation Dionisio Sese and Tobita [25]. Malondialdehyde (MDA) concentration in the leaves and roots had been measured. First of all, grinding of 0.5 g leaves and roots sample was performed in TCA solution. The two readings had been measured at 532 nm and 600 nm on spectrophotometer for the measurement of MDA in the roots and shoots of plants.

A 50 mg sample was taken and 3.0 mL solution of phosphate buffer was included and centrifuged for 30 min (6000× *g* 4 °C) for 30 min for estimation of H_2_O_2_. The titanium sulfate 1.0 mL (0.1%) was mixed in supernatant again and centrifugation carried out (6000 rpm at 4 °C) for 20 min. The absorption of supernatants was determined at 410 nm and calculation of H_2_O_2_ 0.28 µmol^−1^ cm^−1^ had been used as an extinction coefficient. The activities of superoxide dismutase (SOD), ascorbate peroxidase (APX), catalase (CAT) and peroxidase (POD) had been determined by spectrophotometer (Halo DB-20/DB-20S, Dynamica Company, London, UK). The grounding of samples was conducted by the help of pestle and mortar in liquid nitrogen after standardizing at 0.05 M phosphate buffer. The centrifugation was also carried out (12,000 rpm at 4 °C) for 10 min and supernatant collected. POD and SOD activities determined by the method suggested by Zhang [26], while the evaluation of CAT was conducted using method narrated by Aebi [27] and APX by Nakano and Asada [28].

### 2.6. Cadmium Assessment in Plants

For the assessment of Cd roots and shoots samples were grinded then digested using HNO_3_–HClO_4_ (3:1, *v*/*v*) and kept the samples for overnight. After this, treated samples 5.0 mL were taken and added more HNO_3_ put on a hot plate for digestion. Then Cd concentration had been measured by means of atomic absorption spectrophotometer (novAA^®^ 350–Analytik Jena, Neckarsulm, Germany).

### 2.7. Statistical Analysis

All the collected data had been subjected to two-way analysis of variance test (ANOVA) for assessing the consequence of P-fertilizers in addition to Cd concentration impact on plant biomass in addition biochemical variables. Tukey’s post hoc test was applied. SPSS (Statistical software, (SPSS, version 23.0 for windows; IBM Corporation, Armonk, NY, USA)) had been used for Statistical analysis.

## 3. Results

### 3.1. Calculation of P-Fertilizer Effects on Biomass and Growth

**Figure 1:**
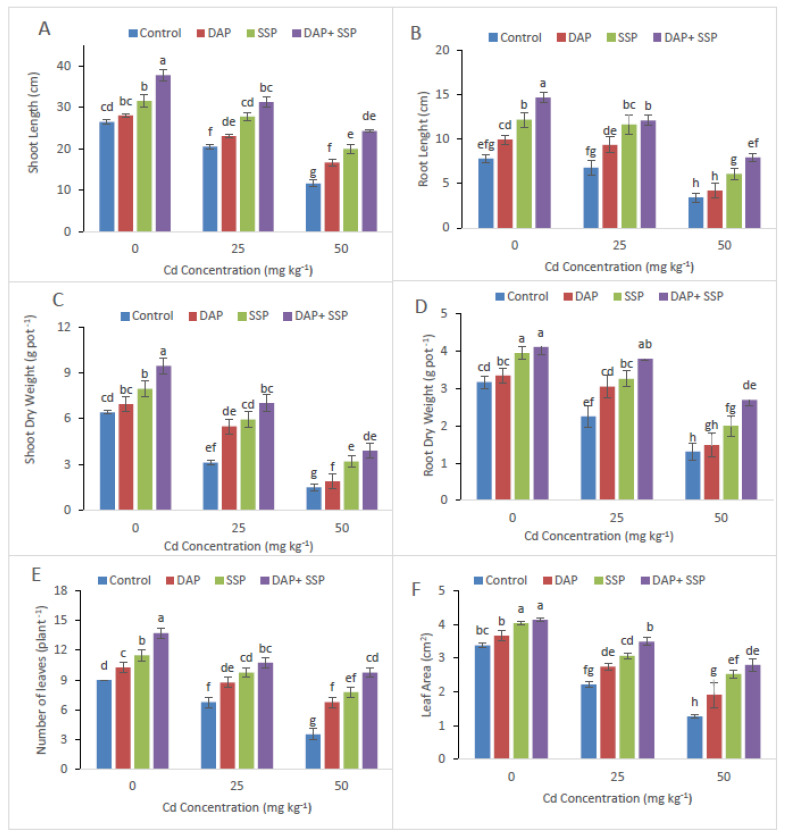
Cadmium stress (0, 25, 50 mg kg^−1^) altered shoot length (**A**), root length (**B**), shoot dry weight (**C**), root dry weight (**D**) number of leaves (**E**) and leaf area (**F**) of *S. nigrum*. Significant increases were observed on all parameters with the increase of P application levels (0–0, 100–0, 0–100, 50–50 mg kg^−1^). The significant difference between the values is of *p* < 0.05 which is shown by different letters.

In control group, visible stunted growth and leaves chlorosis was observed while no such symptom was seen in P-Fertilizer Fed group (Figure 1). The Root-shoot dry mass, number of leaves per-plant and root-shoot length of the *S. nigrum* plants remarkably increased in P-fertilizers treatments (Figure 1). Maximum plant growth was observed in combine application of DAP + SSP-Fed plants at lower Cd level (25 mg kg^−1^) compared to single DAP-treated, SSP-Fed and control plants. Additionally, more number of leaves (70%) had been observed in DAP + SSP-Fed compared to DAP, SSP-Fed plants (Figure 1).

### 3.2. Calculation of P-Fertilizer Effects on Gas Exchange Traits and Chlorophyll Contents

**Figure 2:**
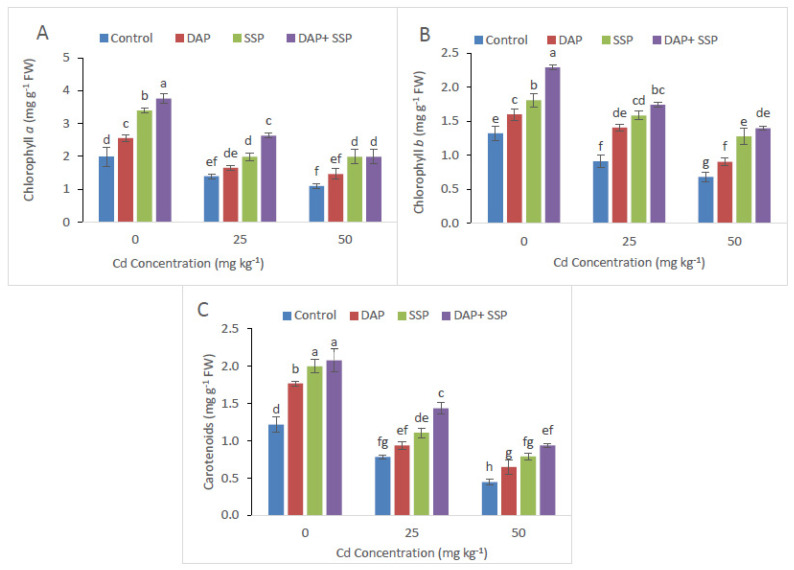
Cadmium stress (0, 25, 50 mg kg^−1^) altered chlorophyll a (**A**), chlorophyll b (**B**) and carotenoid (**C**) of *S. nigrum*. Significant increases were observed on all parameters with the increase of P application levels (0–0, 100–0, 0–100, 50–50 mg kg^−1^). The significant difference between the values is of *p* < 0.05 which is shown by different letters.

**Figure 3:**
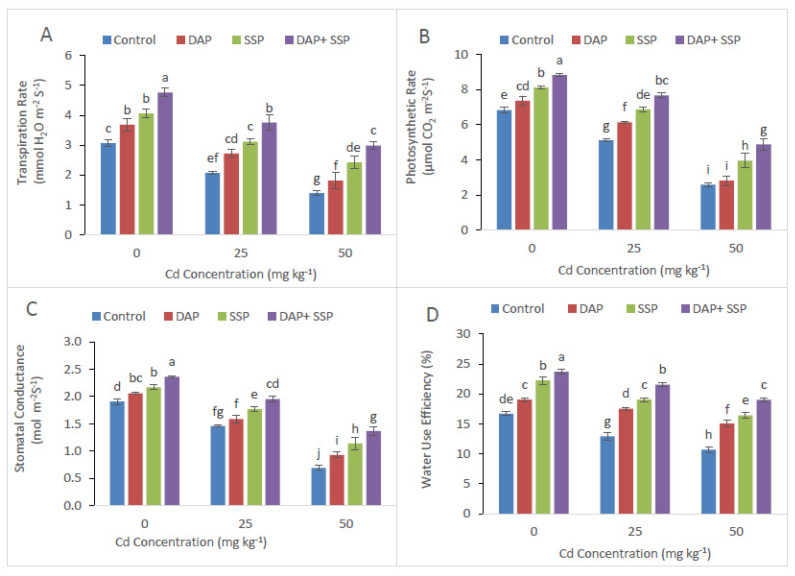
Cadmium stress (0, 25, 50 mg kg^−1^) altered Transpiration rate (**A**), Photosynthetic rate (**B**) Water use efficiency (**C**) and Stomatal conductance (**D**) of *S. nigrum*. Significant increases were observed on all parameters with the increase of P application levels (0–0, 100–0, 0–100, 50–50 mg kg^−1^). The significant difference between the values is of *p* < 0.05 which is shown by different letters.

Distinctive response of gas exchange-attributes along with chlorophyll contents was recorded in combine application DAP + SSP-fed plants and single DAP-treated, SSP-treated plants. In DAP + SSP-fed plants showed significant changes with highest values in carotenoid and Chl *a*, Chl *b*, total Chl content (Figure 2). Significant increase in chlorophyll substances had been noted in DAP + SSP-fed plants at 25 mg kg^−1^ compared to DAP-treated, SSP-treated and control plants. Furthermore, about 70% higher appearance of gas attributes had been recorded in DAP + SSP-fed plants as compared to DAP-treated, SSP-fed plants (Figure 3).

### 3.3. Calculation of P-Fertilizer Effects on EL, MDA and Antioxidant Enzyme Activities

**Figure 4:**
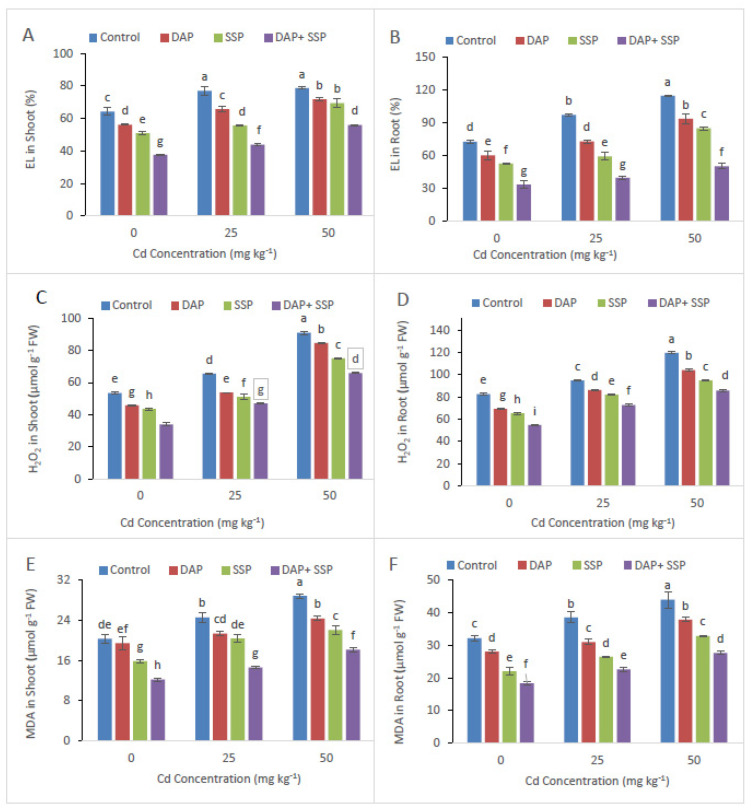
Cadmium stress (0, 25, 50 mg kg^−1^) altered leave leaves EL (**A**), root EL (**B**), leaves, H_2_O_2_ (**C**), root H_2_O_2_ (**D**), MDA (**E**) and root MDA (**F**), of *S. nigrum*. Significant increases were observed on all parameters with the increase of P application levels (0–0, 100–0, 0–100, 50–50 mg kg^−1^). The significant difference between the values is of *p* < 0.05 which is shown by different letters.

**Figure 5:**
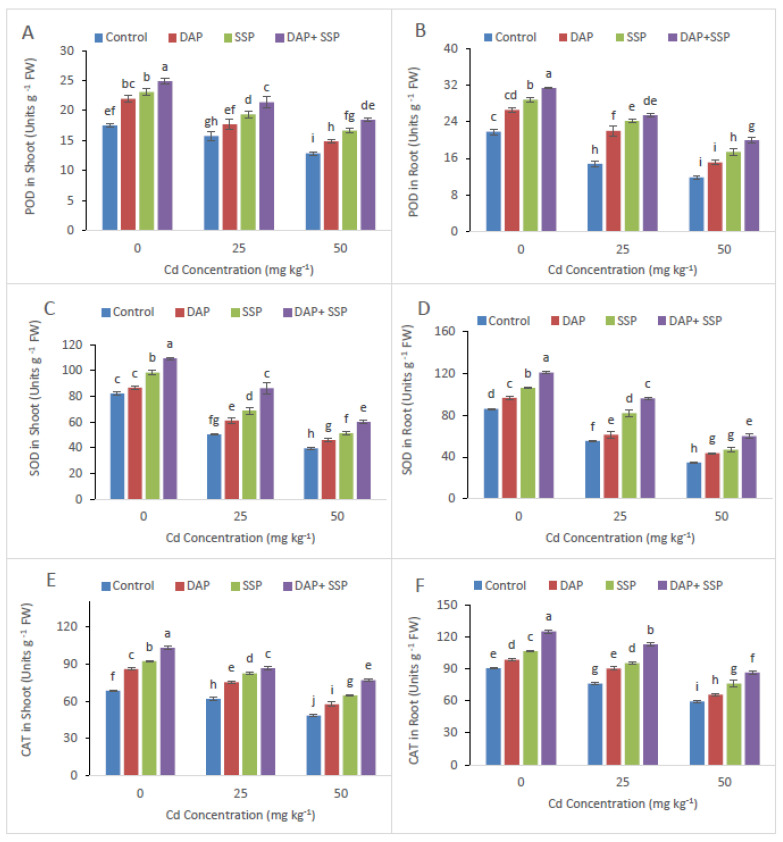

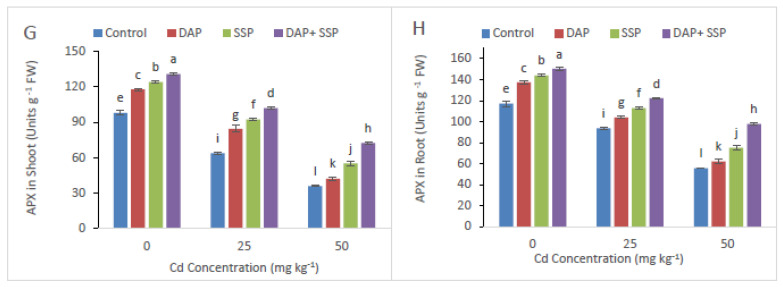
(Cd) stress (0, 25, 50 mg kg^−1^) altered leaves POD (**A**), root POD (**B**) SOD (**C**), root SOD (**D**), leaves CAT (**E**), root CAT (**F**), leaves APX (**G**) and root APX (**H**) of *S. nigrum*. Significant increases were observed on all parameters with the increase of P application levels (0–0, 100–0, 0–100, 50–50 mg kg^−1^). The significant difference between the values is of *p* < 0.05 which is shown by different letters.

Supplementation of P-Fertilizer showed significant reduction in Electrolyte leakage and MDA (Figure 4). Decreases in EL in leaves of DAP, SSP, and DAP + SSP fed plant by 15, 9 and 43%, respectively, likened to the control Plants. Peroxidase (POD), Catalase (CAT), Superoxide dismutase (SOD) and Ascorbate peroxidase (APX), also enzyme activities in *S. nigrum* leaf considerably increased after supplementation of P-fertilizers (Figure 5). Significant increase in CAT, POD, APX and SOD values with the application of P-Fertilizer was observed as contrast to control group.

### 3.4. Calculation of Accumulation of Cd in Plants

**Figure 6:**
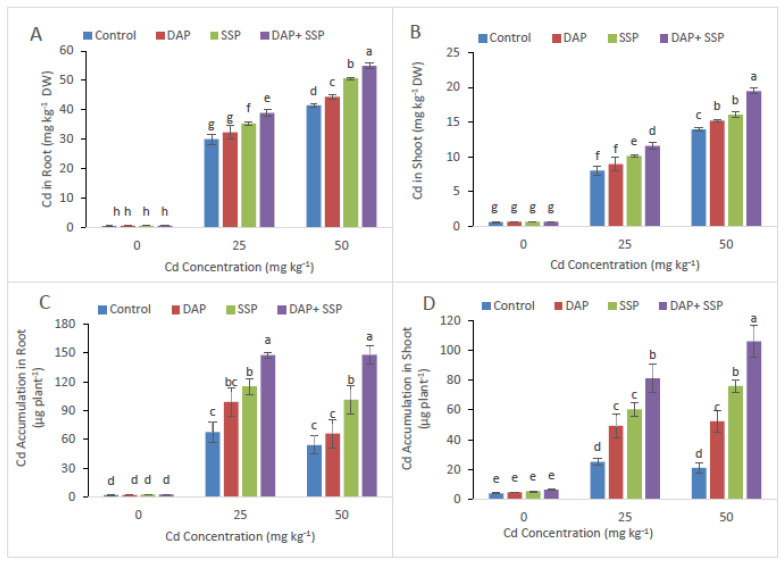
Outcome of Cd stress (0, 25, and 50 mg kg^−1^) and P fertilizers (levels 0–0, 100–0, 0–100, and 50–50 mg kg^−1^ for DAP SSP, and DAP + SSP) on Cd uptake in shoots (**A**), Cd uptake in roots (**B**), Cd accumulation in root (**C**), Cd accumulation in shoot (**D**) of *S. nigrum*. Different letters show a significance difference at *p* < 0.05 along with *n* = 4.

The P-fertilizers optimum induction noticeable improved Cd concentration in root also shoot of *S-nigrum* as contrast to control plants (Figure 6).

## 4. Discussion

It is an established fact that growth of heavy metal stressed plants is greatly affected by the use of fertilizer [29]. In the present investigation, maximum plant development was observed in DAP + SSP-fed plants at 25 mg kg^−1^, compared to DAP, SSP-fed plants (Figure 1). The results of our research are in agreement with the finding of Yu et al. [17] who found that 100 mg kg^−1^ P was the best concentration which promotes the dry biomass of M. jalapa under Cd stress. As Cd levels increased in the growth medium significant reduction was observed in plant growth and biomass [30]. At lower Cd dose (≤12 mg kg^−1^) *S. nigrum* plant showed normal growth but significant reduction observed at Cd leave upper then (24 Cd mg kg^−1^). Wei et al. [31] reported that total root volume, root length, root diameter and root surface area of *S. nigrum* had not affected with soil spiked up to 20 mg kg^−1^ while decreased rapidly when Cd levels higher then up to (40 mg kg^−1^) in growth medium. In this current trial, shoot and root dry weight, shoot-root length and number of leaves plant^−1^ of *S. nigrum* plants remarkably increased as increasing quantity of P-fertilizers (Figure 1). Hammami et al. [9] observed *S. nigrum* more Cd tolerant compared to *Abutilon Theophrastus*, *Portulaca oleracea* and *Taraxacum officinale* weeds. Result of them finding showed dry and fresh mass of roots in addition shoots of all plants decrease as increasing Cd levels in the growth medium; but, lower decline was recorded in *S. nigrum* bio-mass compared to other plants. Present study result indicated that critical concentration of Cd could be reducing by *S. nigrum* with combine application of DAP + SSP fertilizers. In growth medium when P come in contact with Cd they form Cd_3_(PO_4_)_2_ [16], which could mitigate the toxicity of Cd [32] and decrease the accumulation of Cd in plants [21], and promote plant growth Sun et al. [18]. Heavy metal toxicity indicated deleterious impacts on photosynthetic pigment as well as chlorophyll, which are the key element of photosynthesis [33]. For photosynthetic activity, Cd behave as the vigorous inhibitor [5]. The photosynthetic enzyme, Rubisco, is a particularly sensitive process that effects by disturbing Chlorophyll (Chl) loss and its biosynthesis as well as carbon fixation [34]. Jiang et al. [35] found that when maize grown in solution with Cd concentration 6.5 × 10^−1^ mM without P applying negative affect on chloroplast was observed such as chloroplast ruptured and grana destroyed, while external application of applying P reduced the degree of damage the chloroplast. In this present investigation maximum chlorophyll contents were observed DAP + SSP-fed plants at 25 mg kg^−1^, compared to DAP, SSP-fed plants (Figure 1). Under Cd stress, plants have got to induced antioxidant enzymes along with special metabolites for their survival. In fact, it is called general strategy; vital to overcome stress [6]. The *S. nigrum* has the ability to tolerate specific levels of Cd by regulating the antioxidant enzymes. After P-Fertilizer supplementation reduction were observed in MDA and Electrolyte leakage (Figure 4). Significant decreases were seen in EL in leaves at all SSP, DAP as well as DAP + SSP treated plant as a result of 15, 9 and 43%, respectively, over to the control. After supplementation of P-fertilizers significant increased were seen in Ascorbate peroxidase, Catalase, superoxide dismutase, Peroxidase and enzyme activities over control (Figure 5). In experimental group POD value were 12, 23, and 35%, CAT, 34, 51, and 90%, APX 22, 33, and 40% and SOD 33, 45, and 60%, respectively, over control (Figure 3). In tomato plant the visible augmentation was recoded in SOD and POD activities reduction in CAT activity under Cd stress while these enzyme activities were higher in *S. nigrum* the same Cd levels [36]. The best concentration of P-fertilizers significantly improved Cd concentration in root and shoot of *S. nigrum* over control plants (Figure 5). However, additives of P-fertilizers seize oxidative stress and boosted the antioxidants enzymatic activities. Thus, a best concentration and combine supplementations of P-fertilizers would be helpful in phytoremediation of heavy metals by *S. nigrum*.

## 5. Conclusions

Our study concluded that application of P-fertilizers will potentially be a better method to decontaminate the soil with heavy metals through Solanum nigrum L. Significant reduction was seen in plants growth in leaves and roots under Cd stress. P-fertilizers improved plants growth, photosynthesis and antioxidants enzymes activities. Oxidative stress was also observed in those plants which were subjected to Cd stress. However, P-fertilizers supplementation improved plant bio-mass by hampering oxidative stress and boosting antioxidants enzymatic activities. In SSP + DAP-fed plants highest plant growth was observed as compared to SSP, DAP-fed plants. In addition, higher Cd concentrations also found in SSP + DAP-fed plants contrary SSP, DAP-fed plants. Finally, the P-fertilizers supplementation enhanced plants growth and Cd phytoremediation. However, SSP + DAP (50:50) might be productive approach to decontaminate the heavy metals through *Solanum nigrum* L. Thus, a best concentration and combination of supplementations of P-fertilizers would be active in phytoremediation of heavy metals by *S. nigrum*. Future work can be conducted on field level to see the phytoextraction potential of *S. nigrum* at field level.

## Data Availability

Not applicable.
